# Targeting Aging Hallmarks with Monoclonal Antibodies: A New Era in Cancer Immunotherapy and Geriatric Medicine

**DOI:** 10.3390/ijms26114982

**Published:** 2025-05-22

**Authors:** Michele Dal Bo, Marta Gambirasi, Idris Vruzhaj, Erika Cecchin, Abbas Pishdadian, Giuseppe Toffoli, Amin Safa

**Affiliations:** 1 Experimental and Clinical Pharmacology Unit, Centro di Riferimento Oncologico di Aviano (CRO) IRCCS, 33081 Aviano, Italymarta.gambirasi@cro.it (M.G.); idris.vruzhaj@cro.it (I.V.); ececchin@cro.it (E.C.);; 2 Department of Life Sciences, University of Trieste, 34127 Trieste, Italy; 3 Department of Immunology, School of Medicine, Zabol University of Medical Sciences, 98616-15881 Zabol, Iran; 4 Institute of Research and Development, Duy Tan University, Da Nang 550000, Vietnam; 5 School of Medicine and Pharmacy, Duy Tan University, Da Nang 550000, Vietnam; 6 Doctoral School in Pharmacological Sciences, University of Padua, 35122 Padova, Italy

**Keywords:** monoclonal antibodies, aging, cancer, senescence, immunotherapy, SASP

## Abstract

Aging is characterized by a progressive deterioration in physiological function and an increased susceptibility to age-related diseases, such as cancer. Monoclonal antibodies (mAbs) constitute a novel therapeutic approach aimed at addressing aging mechanisms such as cellular senescence, inflammaging, and immunosenescence. This text presents an overview of mAb methods aimed at the markers of aging and their potential application in cancer treatment. The mAbs can be categorized into senolytics, senescence-associated secretory phenotype (SASP) neutralizers, and immune checkpoint inhibitors, each targeting fewer aging-related pathways relevant to cancer therapeutic enhancement than the last. Translating promising preclinical evidence into enhanced efficacy and safety in cancer therapy presents challenges, particularly in older populations. This study examines the therapeutic efficacy of mAbs in the treatment of cancer and age-related disorders, focusing on their current and future roles in oncology practice.

## 1. Introduction

A complex biological process, aging is marked by a gradual reduction in physiological processes that increases an organism’s susceptibility to a range of ailments, including neurological diseases, cardiovascular issues, and different malignancies [1]. Mechanisms fundamental to the aging process include immune system decline (immunosenescence), chronic low-grade inflammation (inflammaging), and cellular senescence [2]. Cellular senescence is the permanent cessation of cell division brought on by stresses, including oxidative stress and DNA damage [3]. Although first acting as a tumor-suppressive mechanism, over time, the buildup of senescent cells causes tissue dysfunction [4]. Together referred to as the SASPs, these cells release a complex mix of pro-inflammatory cytokines, chemokines, and proteases that can upset the tissue microenvironment and induce chronic inflammation [2]. Immunosenescence is the age-related drop in immunological function marked by lower immune surveillance and a higher frequency of infections, cancers, and autoimmune diseases. Changes in both innate and adaptive immunity define this drop and affect vaccination responses, as well as the capacity to react to new antigens [5].

Inflammaging explains the persistent, systemic inflammatory condition seen in the aged that is linked to the development of several age-related disorders. Multiple elements contribute to this ongoing inflammation: alterations in the gut flora, the accumulation of senescent cells, and the constant activation of the innate immune system [6]. Conventional anti-aging treatments have mostly concentrated on lifestyle changes and small-molecule medications meant to control these basic aging processes [7]. Notwithstanding significant progress, many of these approaches have not been very successful in precisely targeting the many processes behind aging [7,8,9,10]. On the other hand, mAbs present a good path for the exact control of aging-related processes. MAbs allow focused intervention with fewer off-target effects than traditional treatments, thanks to their great specificity and affinity [11,12].

In this setting, we suggest a fresh anti-aging mAbs classification system:

**Class One: Senolytics.**

These mAbs are meant to target senescent cells. Targeting particular senescent cell markers, for instance, antibody–drug conjugates (ADCs), has shown effectiveness in eradicating senescent cells, hence reducing their negative impact on tissue homeostasis [13].

**Class Two: SASP Neutralizers.**

This class comprises mAbs that neutralize SASP components, including pro-inflammatory cytokines IL-6 and IL-8, thereby reducing the chronic inflammation related with aging [14,15].

**Class Three: Immunity Rejuvenators.**

These mAbs target immunological checkpoints to revitalize an aged immune system [16]. Anti-PD-1 antibodies, for example, have been investigated for their ability to boost the immune system in the aged, hence enhancing responses to cancer and infections [17,18]. Recent studies have highlighted how modular and combinable targeting approaches can help mAbs to solve the multifactorial character of aging [13,19,20]. Examining these creative ideas helps this review to present a thorough study of how mAb-based treatments might transform geriatric medicine and provide fresh ideas to improve healthspans and fight age-related disorders.

### 1.1. MAbs: Mechanisms, Strategies, and Pathologies Addressing Aging Hallmarks Interpretive Meaning

**Figure 1:**
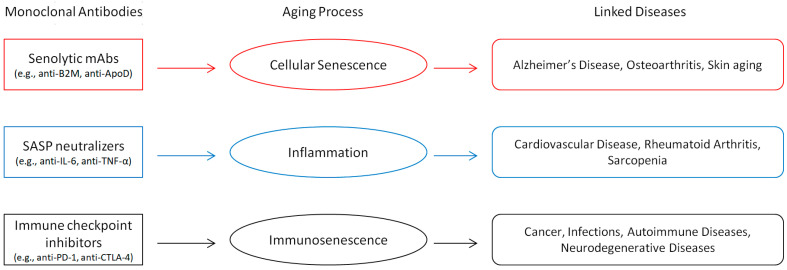
MAb strategies targeting aging processes and related diseases.

Commonly known as the hallmarks of aging, a network of linked biochemical pathways drives the aging process [21]. These mechanisms help to explain the slow degradation of tissue integrity and systemic performance that is connected with aging. mAbs have emerged as revolutionary therapeutic agents, transforming the treatment of cancer, autoimmune diseases, and viral infections [22,23,24]. The evolution of mAbs specifically targeting important hallmarks is described in this section, together with its relevance in the treatment of age-related diseases. Figure 1 presents a comprehensive review of various mAb techniques, classifying them into senolytics, SASP neutralizers, and immune checkpoint inhibitors, while delineating their targets within aging processes and associated disorders.

This figure delineates the three principal categories of mAb strategies senolytic mAbs, SASP neutralizers, and immune checkpoint inhibitors, along with their specific targets in aging processes, including cellular senescence, inflammaging, and immunosenescence. It underscores the prospective therapeutic uses of these tactics for age-related ailments, encompassing Alzheimer’s disease (AD), osteoarthritis, cardiovascular disease, cancer, and neurological disorders.

#### 1.1.1. Cellular Senescence Mechanism

An irreversible cell-cycle arrest defines the stress-induced disorder known as cellular senescence [25]. Originally helpful in preventing the growth of damaged or precancerous cells, senescent cells often gather as one ages. They emit a diverse array of pro-inflammatory chemicals that establish a chronic inflammatory milieu, contributing to tissue dysfunction [26] and progressively correlating with numerous age-related illnesses.

#### 1.1.2. Senescence and Its Role in Aging and Oncology: Therapeutic Implications

Cellular senescence, a process in which cells irreversibly cease division due to stress or damage, is a vital mechanism that can both safeguard against and facilitate cancer development. Senescence fundamentally functions as a protective mechanism, inhibiting the unchecked development of potentially malignant cells. This condition may also serve as a double-edged sword [27]. Senescent cells retain metabolic activity and release a range of chemicals, together termed the SASPs, which can affect their surroundings in diverse manners. In cancer, this secretory phenotype may serve a dual function: initially contributing to tumor suppression, but potentially facilitating their growth over time [28].

The relationship between senescence and cancer is intricate. Oncogene-induced senescence (OIS), instigated by mutations in genes such as RAS or BRAF, exemplifies a scenario where senescence functions as an initial tumor-suppressive mechanism, inhibiting the proliferation of precancerous cells [29]. Conversely, certain substances released by senescent cells can facilitate tumor proliferation and spread. Cytokines such as IL-6 and IL-8, prevalent in the SASP, can promote cancer cell proliferation, motility, and angiogenesis, hence aiding tumor dissemination [30].

Cancer therapies, such as chemotherapy and radiation, can provoke senescence in cancer cells, resulting in therapy-induced senescence (TIS) [31]. The buildup of senescent cells in non-cancerous tissues frequently leads to detrimental effects, despite the prevention of future tumor growth. This encompasses diminished tissue function, fragility, and the expedited aging observed in numerous cancer survivors, especially among older individuals. Senescence may hinder the immune system’s capacity to eliminate cancer cells, as the immune response might be weakened by the persistent inflammatory signals generated by senescent cells within the tumor microenvironment (TME) [32].

Due to these complications, addressing senescence in cancer therapy has emerged as a focal point of considerable attention. A promising strategy involves the application of senolytic treatments, designed to specifically eradicate senescent cells [33]. Eliminating these cells from the TME could diminish inflammation and immune evasion, hence enhancing the efficacy of other cancer therapies. Another method, senomorphic treatment, aims to regulate the SASP by modulating the secretory factors without eradicating the senescent cells, thereby maintaining their tumor-suppressive advantages while mitigating their detrimental effects [28].

**Strategies for MAbs:**

- **Senolytic ADCs:** Certain mAbs are being developed as transporters for lethal medicines, mostly targeted at markers, like Apolipoprotein D (ApoD), that are common on the surfaces of senescent cells. Senolytic ADCs are designed to destroy harmful cells while maintaining healthy tissue; these senolytic ADCs help to restore the local tissue balance and thereby reduce the senescence-associated secretory phenotype [13].
- **Neutralizing Antibodies Against SASP**: Another approach is concentrating on particular components of the SASP, including IL-6, IL-8, and TNF-α; in other words, neutralizing antibodies against SASPs. By reducing local inflammation, neutralizing pro-inflammatory cytokines may help to either delay or stop tissue deterioration in aging organs [34,35,36].

**Applications in Disease** **:**

- **AD**: Senescent glial and neuronal cells are thought to play a role in neuroinflammation and cognitive deterioration. Preliminary investigations of senolytic antibodies indicate their potential to diminish neurodegenerative disease in preclinical settings [37].
- **Osteoarthritis**: Inflammatory senescent cells within the joints are associated with cartilage degradation and discomfort. Senolytic therapies have demonstrated encouraging outcomes in reducing disease severity and enhancing joint health in animal research [38,39,40].
- **Skin Aging**: The accumulation of senescent fibroblasts and keratinocytes is associated with observable aging indicators, such as diminished skin suppleness and the formation of wrinkles. The precise removal of these cells may create new opportunities in dermatological rejuvenation therapy [41,42,43].

**Developmental Status**: Senolytic MAbs, especially those formulated as ADCs, remain in the nascent phases of clinical research; however, an increasing array of preclinical studies reinforces their therapeutic potential [44]. Multiple candidates are presently undergoing safety and efficacy evaluations in models of age-related diseases [45].

### 1.2. Inflammaging Mechanism

Inflammaging denotes the persistent, subclinical inflammatory condition that gradually arises with advancing age. In contrast to acute inflammation, which subsides following injury or illness, inflammaging is persistent and systemic [6]. The phenomenon is chiefly propelled by elevated circulation concentrations of pro-inflammatory cytokines, including interleukin-6 (IL-6), interleukin-1 beta (IL-1β), and tumor necrosis factor-alpha (TNF-α) [46]. These mediators are pivotal in facilitating tissue damage, immunological dysregulation, and metabolic imbalance and are significantly associated with the initiation and advancement of several age-related illnesses [46].

**Strategies for MAbs:**

- **Anti-IL-6 Receptor Antibodies**: Therapeutic antibodies like Tocilizumab and Sarilumab inhibit the IL-6 receptor, thereby obstructing a crucial inflammatory signaling pathway. Initially formulated for rheumatoid arthritis, these antibodies are currently being explored for their potential in several inflammation-related illnesses linked to aging [47].
- **Anti-TNF-α Antibodies:** MAbs such as Infliximab and Adalimumab directly inhibit TNF-α, a principal regulator of systemic inflammation. Their immunomodulatory effects have been well established in the management of autoimmune disorders, and their significance in aging-related inflammation is becoming increasingly evident [48].

**Applications in Disease:**

- **Rheumatoid Arthritis (RA)** exemplifies inflammaging, characterized by chronic cytokine activity that leads to joint deterioration and systemic inflammation. Anti-cytokine medications, such as mAbs, have revolutionized treatment, demonstrating their clinical effectiveness in reducing the inflammatory burden [49].
- **Cardiovascular Disease (CVD):** Chronic inflammation exacerbates endothelial dysfunction and plaque development, hastening atherosclerosis in the elderly. Recent results indicate that anti-cytokine mAbs may provide vascular protection by attenuating inflammatory pathways implicated in the etiology of cardiovascular disease [50].
- **Sarcopenia:** Muscle atrophy and weakness associated with aging, generally referred to as sarcopenia, have been correlated with increased inflammatory cytokines [51]. Despite the preliminary nature of clinical data, cytokine-targeting mAbs may offer potential in maintaining muscle mass and function in the aged.

**Developmental Status**: Numerous mAbs directed against IL-6 and TNF-α have received FDA approval for the treatment of autoimmune and inflammatory disorders [52,53,54,55]. Current translational research is investigating the potential repurposing or adaptation of these medicines for the treatment of systemic inflammation in elderly populations. Subsequent trials will be essential in assessing their long-term safety and efficacy in geriatric settings.

### 1.3. Immunosenescence Mechanism

Immunosenescence denotes the age-related deterioration of immunological functionality, marked by both functional and structural alterations within the innate and adaptive components of the immune system. This reduction leads to heightened susceptibility to infections, reduced vaccine efficacy, elevated cancer rates, and an overall deterioration in immunological surveillance. The characteristics of immunosenescence encompass diminished T-cell receptor diversity, the accumulation of fatigued T-cells, compromised antigen presentation, and a pro-inflammatory alteration in the immunological environment [56].

**Strategies for MAbs:**

- **Checkpoint Inhibition**: A promising strategy to mitigate immunosenescence is the application of mAbs that target immunological checkpoint proteins, including Programmed Death-1 (PD-1) and Cytotoxic T-Lymphocyte-Associated Protein 4 (CTLA-4). Inhibitory receptors are frequently increased on T-cells in elderly persons, leading to functional fatigue. Immunological checkpoint inhibitors, like Nivolumab (anti-PD-1) and Ipilimumab (anti-CTLA-4), have been shown to be effective in oncology by reactivating antitumor immunity and are currently being investigated for their potential to revitalize immunological responses in aging populations more generally [16,57].

**Applications in Disease:**

- **Cancer:** The age-associated deterioration of immune surveillance promotes tumor evasion and advancement. By obstructing inhibitory checkpoints, mAbs can reinstate cytotoxic T-cell functionality and enhance tumor identification, rendering them a formidable choice for cancer therapy in elderly patients [16].
- **Infectious Diseases**: Elderly adults demonstrate diminished responses to infections and vaccinations. Preliminary studies indicate that altering checkpoint pathways may augment pathogen-specific T-cell responses and increase outcomes in viral and bacterial infections in the elderly [58].

**Developmental Status**: Checkpoint inhibitors have received regulatory approval for many malignancies and are currently employed in standard clinical practice. Nonetheless, their extensive application in immunological dysfunction associated with aging is still in preliminary phases. Current trials are assessing the safety and efficacy of these medicines in reversing parts of immunosenescence while minimizing excessive autoimmunity, especially in frail or comorbid elderly individuals [59,60].

### 1.4. Disruption of Proteostasis Mechanism:

A characteristic of aging is the gradual decline of proteostasis: the biological ability to sustain appropriate protein folding, repair, and destruction. As organisms age, the quality control mechanism becomes progressively overwhelmed, leading to the buildup of misfolded, aggregated, or damaged proteins [61]. These protein aggregates can disrupt vital cellular functioning and are significantly associated with the etiology of neurodegenerative disorders, including Alzheimer’s and Parkinson’s [62].

**Strategies for MAbs:**

- **Anti-Amyloid MAbs**: Numerous mAbs have been engineered to identify and attach to aggregated forms of amyloid-beta (Aβ), a peptide pivotal to the pathogenesis of AD. Aducanumab and Lecanemab are agents engineered to promote the immune-mediated removal of Aβ plaques from the brain, potentially alleviating subsequent neurodegeneration [63,64]. These antibodies operate by either enhancing phagocytosis via microglial activation or by directly obstructing plaque formation and dissemination.
- **Alzheimer’s and Parkinson’s Diseases: Clinical Trials and Therapeutic mAbs:**

Recent advancements in immunotherapeutic strategies have produced tailored mAbs against pathogenic protein aggregation in neurodegenerative diseases. In AD, three anti-amyloid antibodies have received FDA approval: aducanumab (2021), lecanemab (2023), and donanemab (2024) exhibit a 23–35% reduction in clinical decline, albeit with differing risks of ARIA [65,66,67]. Immunotherapies for Parkinson’s disease (PD) that target α-synuclein have inconsistent results: prasinezumab exhibited positive signals in secondary endpoints despite failing to meet primary outcomes in phase 2b [68], but cinpanemab did not demonstrate a clinical benefit [69]. This is summarized in Table 1. This changing environment underscores both the potential and difficulties of mAbs in age-related neurodegeneration.

**Applications in Disease:**

- **AD:** The accumulation of amyloid-beta plaques is a characteristic hallmark of AD. Anti-Aβ antibodies seek to diminish amyloid accumulation and maintain cognitive function in the initial phases of the disease. Although clinical trials have shown reductions in the plaque burden, the degree of functional improvement is limited, and factors like patient selection, the timing of intervention, and safety concerns (e.g., amyloid-related imaging abnormalities, or ARIA) continue to influence their therapeutic application [70].

**Developmental Status:** Anti-amyloid mAbs, including Aducanumab and Lecanemab, have obtained accelerated approval from the U.S. Food and Drug Administration for the treatment of early-stage AD [71,72]. Nonetheless, their therapeutic efficacy remains questionable, especially considering the substantial expenses, diversity in patient responses, and the potential for treatment-related problems. Consequently, these interventions continue to be a central topic of research and discussion in the domain of neurodegenerative aging.

### 1.5. Advanced Glycation End-Products (AGEs)

AGEs are a category of diverse compounds generated via non-enzymatic interactions between sugars and proteins or lipids. Over time, these glycated molecules accumulate in organs, modifying protein structure and function while exacerbating oxidative stress and inflammation. AGE buildup is an acknowledged factor in cellular aging and has been associated with the pathogenesis of various degenerative disorders, especially in metabolically impaired individuals [73].

**MAb Approaches:**

- **AGE-Targeting Antibodies:** Innovative MAbs, such as SIWA318H, have been designed to preferentially identify and bind to AGE-modified proteins, facilitating their immune clearance. This strategy seeks to alleviate the pro-inflammatory and pro-fibrotic impacts of AGEs, thereby diminishing their role in chronic tissue damage. By mitigating the burden of AGEs, these antibodies may facilitate the restoration of homeostasis in aged tissues [74].

**Applications in Disease:**

- **Complications Associated with Diabetes**: AGEs are pivotal in the development of diabetic microvascular and macrovascular problems, such as retinopathy, nephropathy, and atherosclerosis. Mitigating AGE buildup may reduce vascular damage and enhance long-term results in diabetic patients [75].
- **Chronic Kidney Disease (CKD):** AGE accumulation in renal tissues leads to fibrosis and deteriorating kidney function, particularly in elderly persons with comorbidities. MAb treatments aimed at advanced glycation end-products may present an innovative approach to decelerate chronic kidney disease progression by diminishing fibrotic signaling and maintaining nephron integrity [76].

**Developmental Status**: MAbs directed against AGEs are currently in preclinical research and signify a viable therapeutic approach [74,77]. As interest in glycation biology and its association with aging increases, these therapies are being assessed for their potential to prevent or reverse AGE-related dysfunction in metabolic and renal disorders.

### 1.6. Dysregulated Nutrient Sensing

Dysregulated nutrition sensing is a fundamental characteristic of aging, primarily caused by the persistent overactivation of growth-promoting signaling pathways, notably the insulin/IGF-1 signaling (IIS) axis and the mechanistic target of rapamycin (mTOR) pathway [78]. Although these pathways facilitate anabolic processes and tissue development throughout youth, their continued activation in later life has been linked to heightened risks of cancer, metabolic disorders, and reduced longevity. The age-related transition to a pro-growth state compromises cellular maintenance and repair, hindering autophagy, proteostasis, and metabolic balance [9]. For further details, see Table 2.

**Strategies for MAbs:**

- **MAbs Against IGF-1R:** Various MAbs, including Dalotuzumab, Cixutumumab, and Teprotumumab, have been engineered to obstruct IGF-1 receptor (IGF-1R) signaling [79,80,81]. Initially developed as anti-cancer medicines, these antibodies may be repurposed to mitigate excessive growth signals, decrease cellular proliferation, and potentially activate longevity-related mechanisms, such as autophagy, in the setting of aging.
- **Antibodies Directed Against mTOR-Associated Nodes**: Although direct mTOR inhibition using mAbs presents a technological hurdle, researchers are investigating indirect approaches that target upstream or downstream modulators of the mTOR system [82]. This involves targeting components of PI3K or S6 kinase (S6K), perhaps enabling the more precise modulation of metabolic activity without completely inhibiting critical mTOR functions.

**Applications in Disease:**

- **Cancer Prevention and Control:** Aberrant IGF-1/mTOR activation facilitates tumorigenesis and advancement [83,84]. Inhibiting IGF-1R signaling may reduce the cancer risk in aging persons or act as a supplementary measure in age-adjusted oncology regimens.
- **Metabolic Syndrome and Type 2 Diabetes:** The modulation of IGF-1 signaling has demonstrated promise for enhancing insulin sensitivity and glucose management in metabolic illnesses that predominantly impact the elderly [85]. Antibodies directed at this axis may augment or improve current antidiabetic treatments.
- **Neurodegenerative Diseases**: mTOR inhibition is associated with the improved autophagic removal of neurotoxic aggregates, including amyloid-beta and tau [86], suggesting potential advantages in the treatment or prevention of Alzheimer’s and Parkinson’s diseases.

**Developmental Status**: Numerous IGF-1R-targeting antibodies have advanced through initial clinical trials in oncology, demonstrating proven safety profiles in certain patient cohorts. Their application in aging is still at the conceptual and preclinical phases; however, the biological reasoning is persuasive [80,87,88,89]. Continued mechanistic investigations and meticulously structured aging-centric trials will be crucial to substantiate their efficacy in enhancing healthspans and postponing age-associated deterioration.

### 1.7. Side Effects

Senolytic mAbs have demonstrated efficacy in eliminating senescent cells; however, they also exhibit class-specific toxicity. Thrombocytopenia is a notable toxicity, occurring in 15–20% of cases involving B2M-targeting ADCs, due to the shared surface markers between these cells and platelets. Moreover, senolytic mAbs may hinder wound healing by inadvertently removing reparative senescent cells, which are essential for tissue regeneration [90]. mAbs encounter considerable obstacles in elderly persons. Immune ICIs, including anti-PD-1/PD-L1 drugs, have been linked to a heightened occurrence of severe irAEs, such as colitis and pneumonitis, in older patients, affecting overall survival [91]. Cytokine-targeting therapies, such as anti-IL-6 receptor mAbs like sarilumab, have demonstrated efficacy in older rheumatoid arthritis patients; nonetheless, apprehensions over the heightened risk of infections, especially bacterial pneumonia, persist [92]. Immunomodulatory mAbs encounter considerable obstacles in elderly persons. Age-associated pharmacokinetic alterations, such as a 40% elevation in the mAb half-life in octogenarians resulting from diminished renal and hepatic function, may result in extended systemic exposure and an increased likelihood of deleterious consequences [93]. Moreover, paradoxical malfunction of the blood–brain barrier in the aged may concurrently elevate the danger of non-specific neurotoxicity while diminishing target engagement, thereby complicating the therapeutic management of central nervous system disorders [94]. Contemporary mitigation efforts for immunomodulatory mAbs in elderly populations include precision dosage methodologies. The intermittent injection of senolytics has demonstrated the preservation of tissue-reparative cells and the enhancement of tissue repair in elderly adults [95,96]. These advancements highlight the essential requirement for age-specific treatment intervals and reliable biomarkers to effectively implement mAb therapy in geriatric care.

## 2. Senolytic MABs: Targeting Senescent Cells

**Figure 2:**
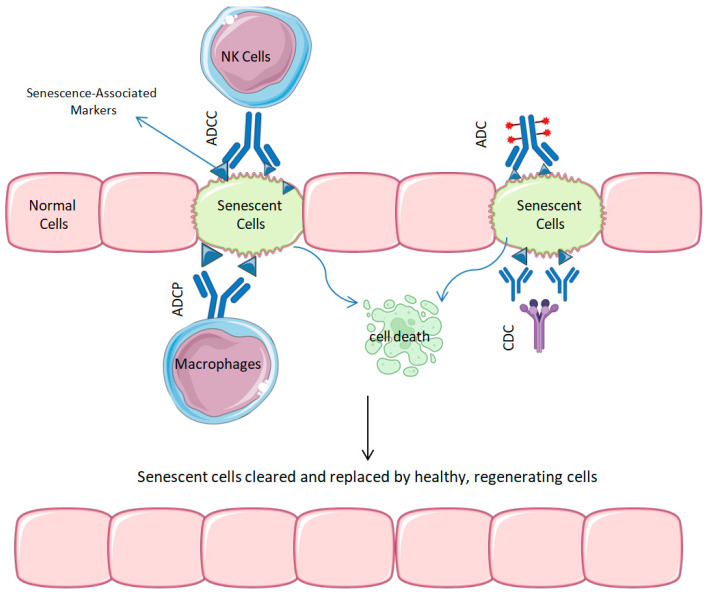
Mechanism of action of senolytic MAbs: Targeting and clearing senescent cells to restore tissue homeostasis. A schematic representation of the mechanisms of action of senolytic mAbs and ADCs. mAbs and ADCs bind to specific surface markers, hence targeting senescent cells (depicted in green). The interaction of MAbs or ADCs initiates immune-mediated responses, including internalization leading to cell death, complement-dependent cytotoxicity (CDC), and antibody-dependent cellular cytotoxicity (ADCC) mediated by natural killer (NK) cells, as well as antibody-dependent cellular phagocytosis (ADCP) mediated by macrophages. Macrophages help maintain tissue homeostasis by eliminating senescent cells, leading to healthier, regenerative tissues.

Biological senescence is a defensive biological mechanism marked by irreversible cell cycle arrest in reaction to several stresses, including telomere shortening, DNA damage, or oncogenic signaling [97]. While senescence initially functions as a tumor-suppressive mechanism, the persistent accumulation of senescent cells over time has been associated with tissue malfunction, delayed regeneration, and the emergence of other age-related illnesses [3]. A significant factor in these harmful effects is the SASP, a pro-inflammatory environment that intensifies chronic inflammation and modifies the local tissue context [13,98]. Recent data indicate that the targeted removal of senescent cells, known as senolysis, may mitigate numerous systemic and localized ailments related to aging. Although small-molecule senolytics, like dasatinib and quercetin, have been extensively researched [42,99,100], mAbs present a highly selective alternative, promising improved precision, fewer off-target effects, and an adjustable design (see Figure 2 for senolytic mAbs mechanism).

### 2.1. ADCs for Senescent Cell Clearance

ADCs exemplify an advanced strategy in senolytic therapy, utilizing the specificity of mAbs to transport cytotoxins directly to senescent cells [13,44]. A significant preclinical advancement features ADC aimed against β2-microglobulin (B2M), a surface antigen that is overexpressed on senescent cells [44]. This ADC, coupled with duocarmycin, specifically triggers apoptosis in senescent cells while preserving healthy, growing cells. In vitro models have shown the effective elimination of senescent populations, whereas in vivo investigations indicate enhancements in tissue integrity and decreases in aging disease indicators.

Various experimental ADC platforms have been developed to identify senescence-associated markers, including uPAR, DPP4 (CD26), and ApoD, with applications spanning skin aging to fibrotic disorders [13,28] (see Table 3). These methods are especially promising in dermatology, as the targeted elimination of senescent fibroblasts has been linked to enhancements in skin thickness, flexibility, and barrier function [101,102]. Notwithstanding their potential, numerous obstacles persist in the translation of ADC-based senolytics to clinical practice. A significant obstacle is the variability of senescent cells among different tissue types and species, which complicates the development of universal surface markers for safe and effective targeting [101,103]. Furthermore, off-target toxicity, payload-associated side effects, and immunogenicity, particularly in older patients with comorbidities, require meticulous assessment in preclinical and clinical investigations [104,105,106,107]. Nonetheless, the increasing complexity of ADC design, coupled with the capacity to tailor mAbs for improved specificity and reduced immunological activation, establishes senolytic mAbs as a persuasive therapeutic category in the nascent domain of geroscience-guided medicine.

### 2.2. The Modulation of the SASP

Besides directly eradicating senescent cells, mAbs provide a potent method for modulating the SASP, a pro-inflammatory mixture of cytokines, chemokines, and proteases that contributes to tissue dysfunction, immune cell recruitment, and chronic inflammation [108]. The targeted disruption of SASP components serves as an adjunct technique to senolysis, especially in regions where the total elimination of senescent cells may be impractical or undesired. An obvious example is ABX-IL-8, a humanized mAb that inhibits interleukin-8 (IL-8) signaling [109]. IL-8 is a significant SASP factor that facilitates inflammation, angiogenesis, and the proliferation of cancer cells. Preclinical findings indicate that ABX-IL-8 can inhibit tumor growth in xenograft animals by interrupting the IL-8 signaling pathway, implying a wider therapeutic potential in mitigating SASP-driven inflammation in aged tissues [110]. Comparable techniques aimed at other SASP components—such as IL-6, MCP-1, and GM-CSF are being explored to diminish tissue-level inflammation while maintaining advantageous senescence effects, including wound healing and tumor suppression [111,112].

### 2.3. Clinical Translation and Challenges

Despite the intriguing potential of senolytic and SASP-modulating mAbs, their clinical advancement faces considerable obstacles. A primary concern is achieving high specificity for senescent cells, due to the absence of universally conserved surface markers and the potential harm to non-senescent cells that have similar antigen expression. This is especially significant in elderly people, as immunological dysregulation and comorbidities may exacerbate undesired consequences [44,111].

A further obstacle is the risk of immunogenicity, particularly with multiple administrations. Although humanization and Fc-engineering have enhanced antibody tolerability, immunological responses to the therapeutic antibodies may diminish efficacy or induce undesirable effects. Furthermore, the establishment of robust clinical outcomes for aging-related therapies, particularly in slowly progressing disorders, continues to pose a regulatory and methodological difficulty [113,114].

Notwithstanding these challenges, the intrinsic specificity, modularity, and tunability of mAbs render them appealing candidates for next-generation senotherapeutics. Progress in antibody–drug conjugate platforms, dual-targeting formats, and biomarker-guided dosing methodologies may expedite their implementation in clinical settings, focusing on maintaining tissue function, mitigating inflammation, and prolonging healthspans in aging demographics [44,115].

**Table 3:** MAb strategies targeting senescent cells.

Strategy	Target(s)	Mechanism	Preclinical/Clinical Status	Ref
ADCs	B2M, Senescent Cell Markers	Delivery of cytotoxic agents to induce apoptosis	Preclinical studies demonstrate senescent cell clearance and tissue rejuvenation.	[44]
SASP Modulation via Cytokine Targeting	IL-8	Antagonism of pro-inflammatory signaling	Investigated in cancer models; potential application in aging under exploration.	[109,110]

## 3. mAbs in Modulating Inflammaging

A prominent biological characteristic of aging is inflammaging, a condition of chronic, low-grade systemic inflammation that arises without evident infection. This condition is propelled by an aggregation of damage-associated molecular patterns (DAMPs), SASP, dysbiosis, and immunosenescence, resulting in the prolonged activation of inflammatory pathways. Increased concentrations of pro-inflammatory cytokines, specifically IL-6, TNF-α, and IL-1, have been repeatedly associated with frailty, cognitive deterioration, atherosclerosis, insulin resistance, and neurodegenerative disorders in the elderly [2,21,116].

MAbs that target these cytokines or their receptors have revolutionized the treatment of autoimmune and inflammatory diseases (see Table 4). Their potential repurposing to modulate inflammaging offers a promising strategy for age-related therapeutic interventions, including conditions such as RA, which highlights the consequences of inflammaging.

- Targeting IL-6:

IL-6 is a multifunctional cytokine that plays crucial functions in both acute and chronic inflammatory reactions. In the realm of aging, consistently high IL-6 levels have been associated with sarcopenia, cognitive decline, and heightened mortality risk. RA, a common autoimmune disorder in the elderly, often shows elevated IL-6 levels [117]. Tocilizumab, a humanized mAb that inhibits the IL-6 receptor (IL-6R), has received approval for the treatment of RA and several inflammatory disorders [118].

- Inhibiting TNF-α:

TNF-α serves as a pivotal mediator within the inflammaging network, facilitating immune cell recruitment, endothelial dysfunction, and insulin resistance [119]. Increased TNF-α levels are commonly found in aging tissues and have been associated with the onset of conditions such as cardiovascular disease, osteoporosis, and neuroinflammation [120]. RA patients also present with elevated TNF-α levels, which contribute to joint degradation and systemic inflammation [121].

MAbs, such as Infliximab and Adalimumab, which directly neutralize TNF-α, are extensively utilized in the treatment of autoimmune disorders, including inflammatory bowel disease and rheumatoid arthritis [122,123]. Initial research indicates that TNF-α inhibition may diminish chronic inflammation and enhance specific indicators of immunological and metabolic health in aged people [124,125]. Nonetheless, apprehensions regarding immunosuppression and the infection risk in elderly individuals necessitate meticulous patient selection and vigilant monitoring.

- IL-1 Inhibition:

IL-1 is pivotal in the initiation and enhancement of inflammatory responses, functioning upstream of both IL-6 and TNF-α [126,127,128]. Increased IL-1 signaling has been linked to various aging-related disorders, such as cardiovascular disease, insulin resistance, and frailty [128]. Anakinra, a recombinant IL-1 receptor antagonist, has been licensed for the treatment of RA and specific autoinflammatory disorders for an extended period. In preclinical and early clinical contexts, IL-1 inhibition has shown the capacity to diminish inflammatory indicators, enhance metabolic function, and mitigate degenerative processes in aged tissues [129]. Anakinra, due to its advantageous safety profile and current regulatory approval, is a viable candidate for repurposing as an anti-inflammaging medication, subject to additional geriatric-specific assessment [130].

### 3.1. The Thbs Pathway Inhibition as a Novel Therapeutic Strategy

In addition to traditional cytokines, current studies have identified new regulators of inflammaging that could be targeted by mAbs. Thrombospondin-1 (Thbs1) is a matricellular protein associated with the aging of hematopoietic stem cells [131]. In mouse models, Thbs1 inhibition has demonstrated the ability to correct age-related hematopoietic deficits and diminish inflammatory signals [132]. The recognition of Thbs1 and analogous non-canonical inflammatory mediators creates opportunities for precision-targeted therapies that extend beyond general cytokine neutralization. MAbs engineered to regulate these pathways may provide enhanced specificity and less immunosuppressive danger, especially in elderly persons with vulnerable immune systems.

### 3.2. Clinical Translation and Challenges

Despite the compelling therapeutic justification for employing mAbs to control inflammaging, numerous translational obstacles persist. Primary among these are apprehensions over safety and immunomodulatory equilibrium in elderly individuals, who exhibit heightened vulnerability to infections, autoimmune exacerbations, and detrimental drug interactions [133,134]. The elevated expense of biologic medicines, especially for long-term or preventive use in extensive aging demographics, presents obstacles to scalability and the sustainability of health systems.

Furthermore, the variability of aging and inflammation hampers patient selection and endpoint definition in clinical trials [135]. Customized biomarkers of inflammaging and tailored dose regimes may be necessary to enhance outcomes. Notwithstanding these obstacles, current research persists in enhancing mAb-based approaches [136], with multiple studies in progress to evaluate their effectiveness in diminishing systemic inflammation, postponing disease onset, and prolonging healthspans in elderly populations.

**Table 4:** MAbs targeting key mediators of inflammaging.

Target Cytokine	MAb	Mechanism of Action	Clinical Application and Status	Ref
IL-6	Tocilizumab	IL-6 receptor antagonist	Approved for rheumatoid arthritis; potential in reducing systemic inflammation in the elderly.	[118]
TNF-α	Infliximab, Adalimumab	TNF-α neutralization	Approved for various inflammatory conditions; studies exploring effects on inflammaging ongoing.	[122,123]
IL-1	Anakinra	IL-1 receptor antagonist	Used in rheumatoid arthritis; research into impact on age-related inflammation underway.	[129]
Thbs1	Not yet developed	Inhibition of Thbs1-mediated inflammaging pathways	Preclinical studies indicate potential in preserving hematopoietic function during aging.	[131]

## 4. Immune Checkpoint Modulation: Rejuvenating the Aging Immune System

As individuals age, the immune system experiences significant restructuring, a process referred to as immunosenescence. This decline is characterized by compromised adaptive immunity, impaired pathogen detection, reduced vaccine efficacy, and heightened vulnerability to infections, cancer, and autoimmune diseases [137]. A concurrent, chronic, pro-inflammatory condition known as inflammaging further undermines immunological resilience. Collectively, these alterations signify a disruption of immunological homeostasis and highlight the necessity for therapies aimed at reinstating immune competence in elderly individuals. One of the most promising therapeutic strategies is the manipulation of immunological checkpoints, which are inhibitory pathways that govern immune activation and sustain self-tolerance. Originally studied in oncology, immune checkpoint inhibition via mAbs is currently being examined for its potential to revitalize the aged immune system (see Table 5).

### 4.1. Understanding Immune Checkpoints in Aging

Essential immunological checkpoints, including programmed cell PD-1 and its ligand PD-L1, are pivotal regulators of T-cell activation [138]. These molecules function as “brakes” to attenuate immune responses and avert excessive inflammation or autoimmunity. In the setting of aging, PD-1 expression is increased on T-cells, especially on CD8+ effector cells, leading to T-cell exhaustion, diminished cytokine output, and compromised immune surveillance [139,140].

**Figure 3:**
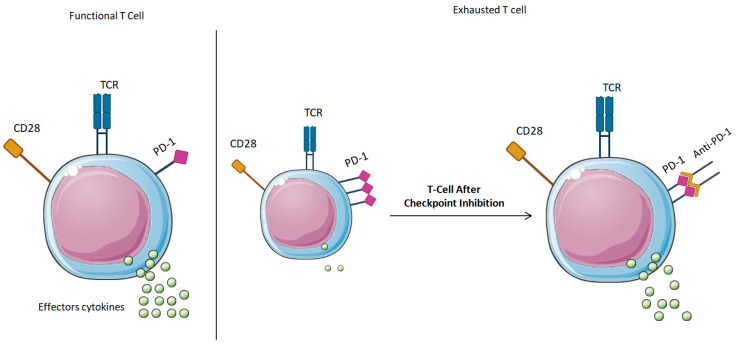
Impact of Immune Checkpoint Inhibition on Aging Immune Function: A Comparative Analysis. A schematic depiction of immunological functionality prior to and subsequent to checkpoint inhibition in aging animals. The diagram contrasts a functional T-cell, a fatigued T-cell marked by elevated PD-1 expression, and a regenerated T-cell following checkpoint inhibition. The checkpoint inhibition mechanism revitalizes T-cell activity, augments cytokine production, and boosts immunological surveillance, hence rejuvenating the aged immune system. The impact of PD-1/PD-L1 inhibition on T-cell activation, memory T-cell regeneration, and immunological functionality is emphasized.

This fatigue phenotype restricts the aging immune system’s capacity to respond effectively to both new infections and neoplastic changes [141]. Checkpoint inhibition, first designed to activate anti-tumor immunity, is currently being investigated as a method to counteract age-related T-cell failure and rejuvenate immunological strength (see Figure 3).

### 4.2. Efficacy of Immune Checkpoint Inhibitors in the Elderly

The clinical evidence regarding immune checkpoint inhibitors (ICIs) in geriatric populations is increasing. A meta-analysis involving more than 17,000 patients, 42% of whom were aged 65 or older, revealed that the overall efficacy of immune checkpoint inhibitors was similar across age demographics [142]. The data indicate that immunosenescence does not universally diminish immune checkpoint inhibitor responses, and that chronological age alone should not exclude the use of immune checkpoint inhibitors in suitably selected patients [142].

Nevertheless, recent findings suggest that patients aged 75 and beyond may demonstrate more inconsistent responses. Factors include elevated PD-1 expression, telomere attrition, and metabolic dysregulation in senescent T-cells may diminish the therapeutic efficacy of immune checkpoint inhibitors in this population. Furthermore, the aging immune system is susceptible to hyperactivation and autoimmunity, heightening concerns for immune-related adverse events (irAEs) in senior patients receiving checkpoint blockade therapy [143].

Checkpoint regulation in aging not only reactivates fatigued T-cells but may also enhance T-cell receptor (TCR) repertoire diversity, augment memory T-cell renewal, and restore homeostatic immune surveillance. The effects of mAbs targeting PD-1/PD-L1 and CTLA-4 suggest their promise as instruments for immunological rejuvenation, contingent upon age-specific biomarkers and functional immune profile guiding their dose, timing, and patient selection [144,145,146].

### 4.3. Novel Targets: GD3 Ganglioside and NKG2D Ligands

Alongside traditional immunological checkpoints, such as PD-1 and CTLA-4, current studies have identified numerous additional targets that may be promising for revitalizing the aged immune system. One specific target is the GD3 ganglioside, a glycolipid often linked to senescent cells [147]. The senescence-associated glycolipid GD3 has emerged as a vital immune checkpoint in the aging process [148]. Recent findings indicate that GD3 on senescent cells interacts with inhibitory Siglec-7/9 receptors on NK cells and macrophages, diminishing phagocytosis and cytotoxicity to facilitate immune evasion [148,149,150]. Anti-GD3 mAbs disrupt GD3-Siglec connections, enhancing the immune-mediated clearance of senescent cells and diminishing systemic inflammation in aged mice. GD3+ senescent CD8+ T-cells accumulate with advancing age and exhibit a correlation with frailty indices, positioning them as a prospective target for immunological rejuvenation [148]. A further intriguing target is the NKG2D ligand family, which is increased in reaction to cellular stress and senescence [151]. The NKG2D receptor on natural killer (NK) cells and CD8+ T-cells recognizes these ligands, which are crucial for the immunological surveillance of stressed or injured cells [152]. Modulating the interaction between NKG2D receptors and their ligands may enhance the immune system’s ability to recognize and eradicate senescent or stressed cells. This method presents a viable strategy for mitigating age-related immunological deterioration and improving the body’s capacity to fight infections, cancers, and degenerative disorders.

### 4.4. Clinical Translation and Obstacles

The regulation of immunological checkpoints and new targets such as GD3 ganglioside and NKG2D ligands offers a unique approach to rejuvenate the aging immune system; nonetheless, numerous clinical obstacles persist. A key worry is the heightened risk of irAEs in aged patients, who exhibit greater susceptibility to autoimmune reactions and other immunological dysregulations [153,154]. This requires meticulous patient selection and vigilant monitoring to prevent the aggravation of concomitant diseases, particularly in vulnerable elderly populations.

The variability of the aging immune system hampers the application of immune checkpoint inhibitors and other targeted medicines. Age-related alterations in immune function can differ significantly among people, necessitating tailored strategies to guarantee maximum efficacy and safety. The creation of indicators to forecast patient reactions to immune modulation is crucial for directing therapy and pinpointing the individuals most likely to gain from treatment.

Additional research is essential for enhancing these medicines, maximizing their application in aging populations, and addressing the problems associated with age-related immunological dysfunction. As we enhance our comprehension of immune aging and the accompanying molecular pathways, the prospect of tailored immunotherapies to revitalize the immune system in the elderly emerges as a progressively feasible approach to augment healthspans and address age-related illnesses.

**Table 5:** Immune checkpoint modulation strategies in aging.

Target	Therapeutic Agent	Mechanism of Action	Clinical Implications	Ref
PD-1	Nivolumab	PD-1 receptor blockade	Approved for various cancers; studies in elderly patients show variable outcomes.	[155]
PD-L1	Atezolizumab	PD-L1 ligand blockade	Used in cancer therapy; potential in targeting senescent cells in aging tissues.	[156,157]
CTLA-4	Ipilimumab	CTLA-4 receptor blockade	Combined with PD-1 inhibitors in cancer; limited data in aging immune modulation.	[158,159]
GD3 Ganglioside	Not yet developed	Targeting senescence-associated immune checkpoints	Preclinical studies suggest potential in enhancing clearance of senescent cells.	[148]

## 5. Molecular and Structural Engineering of MAbs for Aging-Adapted Therapeutics

The advancement of mAbs as therapeutic agents for age-related ailments has distinct obstacles due to the physiological alterations linked to aging. Given that the aged population frequently experiences altered drug metabolism, reduced immune responsiveness, and heightened vulnerability to adverse events, it is essential to create mAbs to tackle these age-specific challenges. This section emphasizes essential tactics in mAb design focused on enhancing its efficacy, safety, and delivery for elderly populations.

### 5.1. Fc Engineering for Improved Durability

Alterations to the fragment crystallizable (Fc) region of mAbs have emerged as a crucial strategy for enhancing their therapeutic durability [113]. These engineering efforts principally aim to prolong the plasma half-life and augment effector functions, both of which are crucial for enhancing the convenience and efficacy of mAb therapy in elderly populations. For instance, the incorporation of particular amino acid changes, such as Q311R/M428E/N434W (REW), has demonstrated a substantial extension of the serum half-life, enhanced mucosal distribution, and enabled fewer frequent dosing schedules [160]. This is especially significant in aged populations, as diminished renal clearance and mobility constraints hinder treatment compliance. Clinical studies indicate that Fc-engineered mAbs, such as satralizumab (anti-IL-6R), may enhance patient adherence via subcutaneous delivery modalities, which provide more convenient administration than conventional intravenous formats [161,162].

### 5.2. Reducing Immunogenicity in Aged Immune Systems

The aging immune system is marked by reduced immunological activity and modified responsiveness, elevating the chance of producing ADAs [163]. Immunosenescence associated with aging results in a two–three-fold increased incidence of anti-drug antibody (ADA) formation in individuals over 65 years of age compared to younger cohorts [5,164]. Modern strategies to mitigate immunogenicity include humanization, deimmunization, and glycoengineering procedures that maintain therapeutic effectiveness while reducing immune recognition. Clinical studies demonstrate that fully humanized mAbs, like adalimumab, exhibit a reduced frequency of anti-drug antibody production relative to chimeric counterparts, which may result in enhanced clinical results [165,166]. The incorporation of immunoregulatory adjuvants (e.g., OPLS) demonstrates significant potential for elderly populations, as it maintains protective immunity while minimizing harmful effects [167]. This balanced strategy is essential for elderly patients who face age-related reductions in immune function yet are still susceptible to hypersensitivity events [5]. Innovative methods integrating glycoengineering with Fc modification may improve tolerance in immunosenescent people; however, cost and accessibility challenges remain in geriatric care environments.

### 5.3. Optimizing for Frail Physiology

Geriatric patients frequently display frailty, polypharmacy, and several comorbidities, necessitating the pharmacokinetic optimization of mAb therapy. Recent studies indicate that 60–70% of patients over 75 encounter treatment-limiting toxicities from typical mAb regimens, necessitating age-adapted formulations. Conventional mAbs formulations, characterized by elevated dosages and intravenous delivery methods, may be unsuitable for elderly populations. Consequently, there is growing interest in lower-dose, long-acting mAb compositions that can alleviate the treatment burden while preserving the therapeutic efficacy [168].

Subcutaneous delivery techniques have been particularly effective in geriatric treatment, with subcutaneously administered rituximab exhibiting comparable efficacy to intravenous therapy while resulting in 40% fewer side effects in patients aged 80 years and older [169]. Bispecific antibodies signify a significant advancement for vulnerable people, concurrently addressing various disease pathways while reducing the medication frequency—an essential benefit for the 30% of aged individuals managing three or more chronic illnesses [170]. These sophisticated formulations include integrated safety characteristics, such as diminished immunogenicity and optimal dosage, tackling significant issues in aging physiology. Significantly, current trials of SC trastuzumab in vulnerable breast cancer patients (NCT04889282) may set new benchmarks for geriatric oncology; however, cost and access obstacles persist as considerable challenges in practical application [171,172].

### 5.4. Targeting Diverse Aging Tissues

The aging process is associated with heightened tissue heterogeneity, complicating the formulation of targeted medicines. Single-cell investigations demonstrate that senescent cell populations can differ by as much as 10-fold across various tissues in older persons [173]. ADCs and bispecific antibodies are promising approaches to address these issues. By integrating the specificity of mAbs with the lethal efficacy of conjugated medicines or dual-targeting strategies, these methodologies facilitate the precise targeting of senescent cells or pathogenic proteins that accumulate in aged tissues [12,105,107]. Recent progress in senescence-associated epitope mapping has revealed many tissue-specific indicators, aiding the creation of next-generation ADCs with improved organ selectivity [174,175]. Nonetheless, obstacles persist in reconciling efficacy with safety, as aged tissues frequently demonstrate heightened susceptibility to off-target effects—an aspect that informs ongoing initiatives to refine therapeutic windows for geriatric applications [176].

### 5.5. Crossing the Blood–Brain Barrier (BBB) in Aging Neurodegeneration

Neurological illnesses common in aging populations, especially AD and Parkinson’s disease, encounter considerable therapeutic obstacles due to deteriorating BBB integrity and penetration difficulties [177]. Receptor-mediated transcytosis strategies have been developed to enhance mAb distribution across the BBB [178]. However, challenges remain in balancing efficacy with safety, as older tissues often exhibit increased vulnerability to off-target effects, which influences current efforts to optimize therapeutic windows for geriatric use. The issues are exacerbated by age-related alterations in the blood–brain barrier, which diminish mAb transport by 40–60%, hence complicating therapy efforts for older persons [66]. Transferrin-receptor-mediated transcytosis methods demonstrate potential, as BrainShuttle^®^-facilitated trontinemab achieves a five-fold increase in CNS delivery compared to traditional mAb in monkey models [179]. Nonetheless, clinical translation poses significant challenges, as evidenced by anti-Aβ mAbs: although lecanemab exhibits a moderate therapeutic effect (0.45 CDR-SB improvement), its 26.4% incidence of ARIA in senior individuals underscores the precarious risk–benefit equilibrium in aging brains [180]. Aducanumab’s contentious approval highlights the complexities of age-related vascular susceptibility in amyloid-targeting approaches. Novel approaches, such as GD3-targeted mAbs for α-synuclein clearance in Parkinson’s disease, may present safer profiles by specifically targeting senescent neurons instead of diffuse aggregates [148]. These experiences underscore the necessity for: (1) age-stratified clinical endpoints that extend beyond plaque reduction to include measures of functional independence; (2) sophisticated imaging biomarkers to anticipate the ARIA risk; and (3) individualized dosing algorithms that consider blood–brain barrier integrity markers, such as CSF/serum albumin ratios, in elderly populations.

### 5.6. Artificial Intelligence-Driven and pH-Sensitive mAb Design for Enhanced Therapeutic Precision

Artificial intelligence (AI) and pH-sensitive engineering are advancing mAb research for age-related disorders. AI systems are engineering antibodies with enhanced specificity for biomarkers such as glycation end-products and senescent cell indicators, hence enhancing their therapeutic efficacy [181]. Moreover, AI-driven techniques are diminishing immunogenicity risks by optimizing antibody architectures to lessen immune responses in older people [182]. pH-sensitive mAbs utilize the acidic microenvironments of inflamed old tissues, attaining 70% enhanced target engagement compared to standard formats [183]. Nonetheless, obstacles persist, including the restricted accessibility of training datasets for elderly populations in AI models and the variable patterns of tissue acidosis in multimorbid individuals. These concerns are being tackled through projects such as the NIH’s AI-CURE study (NCT05689215).

## 6. Progress in MAb Treatments

MAbs have had considerable progress in recent years. These advancements have resulted in enhanced specificity, effectiveness, and safety profiles, along with novel therapeutic opportunities in previously unexplored domains.

### 6.1. Improved Specificity and Diminished Adverse Effects

The advancement of second-generation anti-amyloid mAbs, such as aducanumab, lecanemab, and donanemab, represents a notable achievement in the management of AD [66,184,185]. These mAbs enhance efficacy in diminishing Aβ deposition and decelerating cognitive decline by preferentially targeting aggregated forms of Aβ. This increased affinity for aggregated Aβ leads to more efficient plaque clearance, an essential process in alleviating AD-related pathology [186]. Notwithstanding these gains, problems persist, especially amyloid-related imaging abnormalities (ARIAs), which have been noted with specific anti-Aβ medications [187]. Amyloid-related imaging abnormalities (ARIAs) are MRI-detected adverse effects observed in individuals on anti-Aβ therapy [187]. ARIA-E (edema/effusion) is triggered by transient neuroinflammation during amyloid clearance, whereas ARIA-H (microhemorrhages/hemosiderosis) results from vascular amyloid removal [188]. The frequency of issues among APOE ε4 carriers underscores the necessity for meticulous safety oversight in clinical practice [189]. The unfavorable consequences associated with ARIAs highlight the necessity for continuous enhancement in mAb design to augment safety profiles and mitigate potential dangers for elderly patients, who are more susceptible to these issues. As research advances, the equilibrium between efficacy and safety will remain a fundamental concern in the optimization of mAb therapy for neurodegenerative disorders. Second-generation anti-Aβ mAbs have included structural enhancements to reduce unwanted effects. Lecanemab preferentially binds to soluble Aβ protofibrils instead of vascular amyloid, hence decreasing the incidence of ARIAs by limiting interactions with delicate cerebral vasculature [66]. Donanemab’s intermittent dosage method, such as ceasing medication upon plaque clearance, significantly reduces ARIA-E (edema) rates in comparison to continuous delivery options [190]. Despite concerns over ARIAs, these advancements illustrate progress in reconciling efficacy and safety for aged people.

### 6.2. Combination Therapies

A novel approach in mAb therapy entails the integration of mAbs with additional therapeutic modalities to produce synergistic effects. A promising strategy involves the integration of anti-Aβ mAbs with anti-tau treatments or anti-inflammatory medicines. This technique addresses various clinical facets of AD, encompassing tau protein buildup, neuroinflammation, and amyloid plaques [191].

This multifaceted strategy may improve the overall therapy efficacy and offer a more holistic answer to Alzheimer’s and other neurodegenerative illnesses. Currently, these combination medicines are being rigorously studied, with current clinical trials assessing their potential to markedly enhance patient outcomes and impede the advancement of these severe diseases.

### 6.3. Expanding Applications Beyond Neurodegenerative Diseases

Although mAbs have primarily been linked to neurological illnesses like AD, their applicability is broadening to encompass other age-related ailments. Anti-CD20 mAbs have shown effectiveness in the treatment of hematologic malignancies, including non-Hodgkin lymphoma and chronic lymphocytic leukemia, as well as certain autoimmune illnesses such as rheumatoid arthritis [192].

Current research is investigating the efficacy of mAbs in cardiovascular diseases, specifically in addressing vascular inflammation and metabolic disorders, including type 2 diabetes [193,194]. This expansion of applications signifies an increasing acknowledgment of the adaptability of mAb therapies and their capacity to address various age-related ailments. As these medicines extend into new applications, mAbs may significantly enhance healthspans and mitigate various chronic illnesses linked to aging. Recent advancements in delivery techniques are markedly improving the efficacy of mAbs in the treatment of age-related disorders, especially those impacting the central nervous system (CNS). A significant obstacle in CNS-targeted therapeutics is the BBB, which restricts the access of most big molecules, including mAbs, to the brain [177]. Mitigating this difficulty has become a primary objective in mAb development, with initiatives aimed at enhancing central nervous system bioavailability.

Roche’s creation of trontinemab, utilizing BrainShuttle technology, exemplifies an inventive resolution to this problem [179]. This method utilizes receptor-mediated transcytosis to enhance the ability of mAbs to traverse the blood–brain barrier, hence augmenting their therapeutic efficacy in neurological disorders such as AD and Parkinson’s disease. These developments offer significant potential for treating age-related neurodegenerative illnesses, because precise drug delivery to the brain is essential for therapeutic efficacy.

### 6.4. Personalized Medicine

The advent of personalized medicine is transforming the domain of mAb therapy, particularly for age-associated conditions. Aging correlates with genomic instability (e.g., telomere degradation, somatic mutations) and proteomic changes (e.g., disrupted nutrient-sensing pathways, inflammaging), which influence drug metabolism and immune responses and target antigen expression [21,195]. APOE ε4 carriers demonstrate expedited amyloid accumulation and modified anti-Aβ mAb clearance, requiring dosage modifications to mitigate the risk of ARIA [189]. Recent progress in biomarker discovery, including SASP profiling and epigenetic clocks [196], facilitates the categorization of aged patients according to their biological age and the degree of immunological senescence. Proteomic markers of inflammaging, such as increased IL-6 and TNF-α, forecast inadequate responses to checkpoint inhibitors in older persons [197], facilitating customized mAb selection. By integrating multi-omics data (genomic, proteomic, metabolomic), doctors can identify the patients who are most likely to benefit from certain mAb.

## 7. Ethical Considerations

- **Informed Consent**

Cognitive impairments common in age-related illnesses, such as AD, might undermine patients’ ability to give informed consent [198]. It is ethically essential for patients or their legal representatives to comprehensively grasp the potential dangers and advantages of mAb therapy.

- **The Evaluation of the Risks and Benefits**

The limited effectiveness and possible side effects of specific mAbs require a thorough assessment of whether the therapeutic advantages outweigh the hazards, especially in at-risk senior demographics. The endorsement of aducanumab for AD has ignited discussions concerning its clinical significance and ethical rationale.

- **The Allocation of Resources**

MAbs therapies, including anti-amyloid medicines and immune checkpoint inhibitors, provide significant cost challenges with limited therapeutic advantages, especially in elderly populations [199]. Health economic assessments indicate inadequate cost-effectiveness for anti-amyloid mAbs, resulting in few improvements in quality-adjusted life years despite substantial expenses [200]. Likewise, checkpoint inhibitors have inconsistent cost–benefit ratios in aged patients, as their efficacy is frequently compromised by immunosenescence and comorbidities [201]. National health expenditures in the U.S. are anticipated to surpass $7.2 trillion by 2031, propelled by swift Medicare expansion and legislative modifications. This transition jeopardizes prioritizing acute care and pharmaceuticals over preventive measures for age-related chronic ailments, with expenditures approaching 20% of GDP [202]. Although anti-TNF-α mAbs in rheumatoid arthritis may save long-term expenses by postponing disability, comparable advantages are lacking in AD, where therapy results remain ambiguous [203]. Ethical frameworks that emphasize patient classification and real-world evidence are essential for aligning resource allocation with societal benefits and ensuring equal access to high-cost medicines.

- **The Utilization of Animals in Research**

The creation of mAbs frequently necessitates comprehensive animal experimentation, a practice that has historically sparked ethical controversy [204]. Concerns regarding animal welfare and the ethical ramifications of exposing animals to tests for human advantage are substantial moral dilemmas. Researchers must evaluate the prospective human advantages of mAb therapeutics against the ethical implications of employing animals in preclinical investigations. Consequently, there is an increasing interest in alternate research methodologies that reduce animal utilization, including in vitro models and computational simulations; nevertheless, these alternatives are not yet entirely sufficient to supplant animal studies in all instances.

## 8. Conclusions

MAbs represent a promising therapeutic strategy for addressing key aging mechanisms—cellular senescence, inflammaging, and immunosenescence—that contribute to aging-related illnesses and cancer. The mAbs enhance immune function, reduce chronic inflammation, and inhibit tumor growth by targeting these mechanisms, thereby providing dual benefits to the aging population. Senolytic mAbs, SASP-neutralizing antibodies, and checkpoint inhibitors are among the leading drugs being investigated for the significant enhancement of cancer therapy in aged patients. Researchers continue to investigate substantial issues pertaining to the effects on an unbalanced immune system, off-target activities, and medication resistance. The future promises the delivery of more selective and efficient medicines through advancements in mAb engineering, particularly via the development of bispecific antibodies and ADCs. As mAbs are further investigated, they may establish foundational therapeutics in geriatric oncology and age-related medicine, offering enhanced prospects for prolonging healthspans and mitigating age-associated illnesses.

## Figures and Tables

**Table 1:** Overview of principal clinical trials for therapeutic MAbs in AD and PD.

Disease	Antibody	Target	Phase	Key Outcomes	Status	Ref
AD	Aducanumab	Aβ aggregates	III	EMERGE demonstrated a 23% reduction in CDR-SB; ENGAGE failed to achieve the primary aim.	FDA-approved (2021); development discontinued in 2024.	[65]
AD	Lecanemab	Aβ protofibrils	III	A 27% reduction in clinical decline on CDR-SB at 18 months; incidence of ARIA-E observed.	FDA-approved (2023); EMA-approved (2025).	[66]
AD	Donanemab	N3pG-Aβ plaques	III	Cognitive decline was mitigated by 35%; elevated occurrence of ARIA-E; EMA declined approval due to risk–benefit apprehensions.	FDA-approved (2024); EMA rejected approval.	[67]
PD	Cinpanemab	α-synuclein	II	No notable impact on illness progression; development has been halted.	Development discontinued.	[69]
PD	Prasinezumab	α-synuclein	IIb	Failed to achieve the primary aim; demonstrated potential therapeutic efficacy in secondary endpoints.	Ongoing evaluation.	[68]

**Table 2:** MAb strategies targeting hallmarks of aging.

Hallmark of Aging	Key Molecular Targets	MAb Strategies	Representative Diseases	Development Status	Ref
Cellular Senescence	B2M, DPP4, IL-6, IL-8	Senolytic ADCs; SASP-neutralizing mAbs	Alzheimer’s, osteoarthritis, skin aging	Preclinical to early phase	[13,14,15]
Inflammaging	IL-6, IL-1β, TNF-α	Cytokine-neutralizing mAbs (e.g., Tocilizumab, Infliximab)	RA, CVD, sarcopenia	Approved in inflammatory disease	[47,48,49,50,51]
Immunosenescence	PD-1, PD-L1, CTLA-4, GD3	Checkpoint inhibitor mAbs (e.g., Nivolumab, Ipilimumab)	Cancer, infection susceptibility	Oncology-approved; aging under investigation	[16,57,58,59,60]
Loss of Proteostasis	Amyloid-β aggregates	Anti-amyloid mAbs (e.g., Aducanumab, Lecanemab)	AD	FDA-approved (limited efficacy)	[63,64,70,71,72]
Advanced Glycation End-products (AGEs)	AGE-modified proteins	Anti-AGE mAbs (e.g., SIWA318H)	Diabetic complications, skin aging	Preclinical	[74,75,76,77]
Dysregulated Nutrient Sensing	IGF-1R, PI3K, mTOR-associated nodes	Anti-IGF-1R mAbs (e.g., Dalotuzumab, Cixutumumab, Teprotumumab)	Cancer, metabolic syndrome, neurodegeneration	Early-phase oncology trials	[79,80,81,82,83,84,85,86,87,88,89]
